# Inhibiting *ex-vivo* Th17 responses in Ankylosing Spondylitis by targeting Janus kinases

**DOI:** 10.1038/s41598-018-34026-1

**Published:** 2018-10-23

**Authors:** Ariane Hammitzsch, Liye Chen, Jelle de Wit, M. Hussein Al-Mossawi, Anna Ridley, Takuya Sekine, Davide Simone, Karen Doig, Alla Skapenko, Paul Bowness

**Affiliations:** 1 Nuffield Department of Orthopaedics, Rheumatology and Musculoskeletal Sciences, University of Oxford, Windmill Road, OX3 7LD Oxford, UK; 2 Department of Nephrology, Klinikum rechts der Isar, Technical University of Munich, Munich, Ismaninger Straße 22, 81675 Munich, Germany; 3 Division of Rheumatology and Clinical Immunology, Medizinische Klinik und Poliklinik IV, University of Munich, Pettenkoferstraße 8a, 80336 Munich, Germany; 4 Present Address: National Institute for Public Health and the Environment (RIVM), Centre for Infectious Disease Control (CIb), Utrecht, The Netherlands; 5 Present Address: Unit for Hematology, Department of Medicine Huddinge, Karolinska Institutet, Stockholm, Sweden

## Abstract

Treatment options for Ankylosing Spondylitis (AS) are still limited. The T helper cell 17 (Th17) pathway has emerged as a major driver of disease pathogenesis and a good treatment target. Janus kinases (JAK) are key transducers of cytokine signals in Th17 cells and therefore promising targets for the treatment of AS. Here we investigate the therapeutic potential of four different JAK inhibitors on cells derived from AS patients and healthy controls, cultured *in-vitro* under Th17-promoting conditions. Levels of IL-17A, IL-17F, IL-22, GM-CSF and IFNγ were assessed by ELISA and inhibitory effects were investigated with Phosphoflow. JAK1/2/3 and TYK2 were silenced in CD4+ T cells with siRNA and effects analyzed by ELISA (IL-17A, IL-17F and IL-22), Western Blot, qPCR and Phosphoflow. *In-vitro* inhibition of CD4+ T lymphocyte production of multiple Th17 cytokines (IL-17A, IL-17F and IL-22) was achieved with JAK inhibitors of differing specificity, as well as by silencing of *JAK1-3* and *Tyk2*, without impacting on cell viability or proliferation. Our preclinical data suggest JAK inhibitors as promising candidates for therapeutic trials in AS, since they can inhibit multiple Th17 cytokines simultaneously. Improved targeting of TYK2 or other JAK isoforms may confer tailored effects on Th17 responses in AS.

## Introduction

Ankylosing Spondylitis (AS) is a chronic inflammatory arthritis affecting the sacroiliac spinal and large joints, with a prevalence of approximately 0.25% in Europe^1^, ultimately causing bony ankylosis, pain and disability^2^. Treatment options have previously been limited to non-steroidal anti-inflammatory drugs and anti-TNF (tumor necrosis factor) biologic agents, with one third of patients not profiting from the latter^3–5^. Therefore alternative treatment options are warranted. More recently antibodies targeting the Interleukin-23/Interleukin-17 (IL-23/IL-17) axis have shown efficacy and, together with findings from genome wide association studies (GWAS) and immune phenotyping data, strongly support a pivotal role for T helper cell type 17 (Th17) responses in AS pathogenesis, and open the road for new drug targets in this pathway^6–10^.

Janus kinases 1/2/3 (JAK1/2/3) and Tyrosine kinase 2 (TYK2), in conjunction with signal transducers and activators of transcription (STAT), are central transmitters of pro- and anti-inflammatory cytokine signals in immune cells and therefore interesting targets for immunomodulation^11^. Other processes depending on JAK-STAT signaling include erythropoiesis, myelopoiesis and platelet production via JAK2, as well as innate anti-viral responses via type I Interferons and Interferon γ (IFNγ)^12^. Tofacitinib, with specificity for JAK1 and JAK3, reduces IL-23 triggered IL-17A secretion from T cells of rheumatoid arthritis (RA) and psoriatric arthritis (PSA) patients *in vitro*^13,14^, and was the first JAK inhibitor to be licensed for treatment of RA in the United States^15,16^. Subsequently Tofacitinib has also been shown to be effective in a phase II clinical trial in AS^17^. Nevertheless Tofacitinib is far less selective than initially thought, especially in CD4+ T cells *in-vitro*^18,19^. Targeting specific JAK could yield selective effects and reduce adverse events, since different JAK are involved in different pro- and anti-inflammatory cytokine signaling cascades. IL-23 signaling, which is required for the stabilization and maintenance of Th17 cells, is thought to be transmitted through STAT3 upon JAK2/TYK2 activation, making inhibitors for these JAK family members most interesting targets for treatment of AS and other Th17-driven diseases^20^. In addition GWAS studies of AS have shown a disease association with a rare single nucleotide polymorphism (SNP) in the *TYK2* gene^21^. Integration of *TYK2* SNP associations across different autoimmune diseases singled out rs3453644 as protective for AS. Homozygocity of the minor allele of this *TYK2* SNP leads to reduced STAT3 phosphorylation upon IL-23 stimulation^22^.

We here demonstrate the *in-vitro* efficacy of JAK inhibition and silencing on Th17 responses from Spondyloarthritis (SPA) patients.

## Results

### JAK inhibitors of different specificities inhibit Th17 responses in CD4+ T cells from patients and healthy controls *in-vitro*

**Figure 1:**
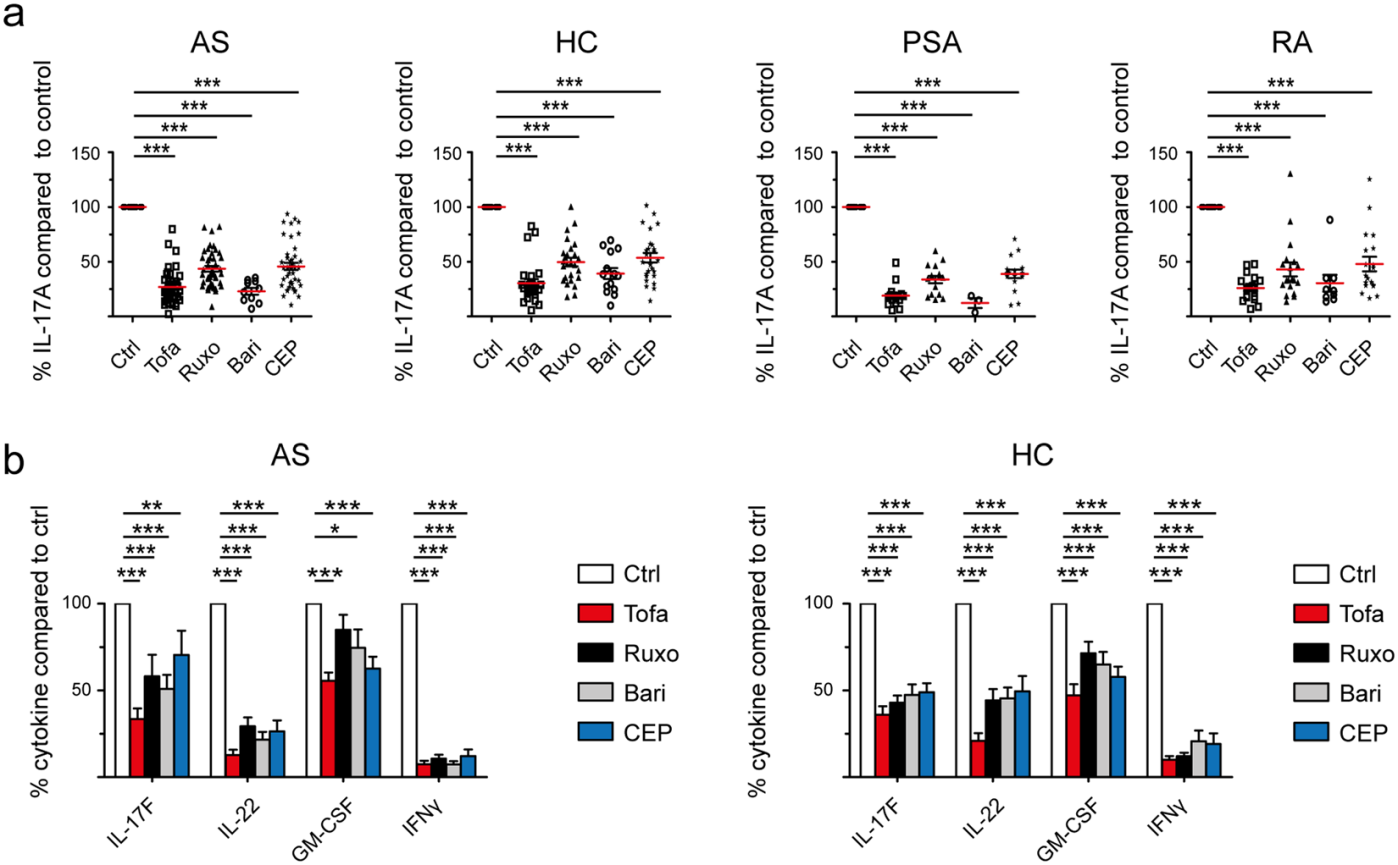
JAK inhibitors inhibit CD4+ T cell “type 17” cytokine production *in-vitro* in Spondyloarthritis, Rheumatoid Arthritis and healthy controls. **(a)** IL-17A secretion from CD4+ T cells cultured under Th17-promoting conditions *in-vitro* in the presence of JAK inhibitors (Tofa, JAK3 > JAK1/2; Ruxo, JAK2 > JAK1; Bari, JAK1/2 > TYK2; CEP, JAK2) from day 0 to 3. Measured by supernatant ELISA and normalized to DMSO control (=100%) on day 3 (no.s AS = 43/Bari = 10, HC = 26/Bari = 14, PSA = 16/Bari = 3 and RA = 18/Bari = 9). **(b)** Inhibitory effects of JAK inhibitors on IL-17F, IL-22, GM-CSF and IFNγ secretion from AS (n = 10 – 8 – 9 – 6 respectively) and HC (n = 10 – 7 – 10 – 10) CD4+ T cells, measured by ELISA as in (**a**) and normalized to DMSO control (=100%). Statistical analysis: mean ± SEM, repeated measures 1-way ANOVA followed by Dunnett’s method for multiple comparisons (**a**) and 2-way ANOVA (**b**) followed by Bonferroni’s method for multiple comparisons.

We first tested four JAK inhibitors of different reported specificity (see Supplementary Table S1) for their ability to inhibit secretion of IL-17A from purified blood CD4+ T cells cultured for 3 days in an initial cohort of 43 Ankylosing Spondylitis (AS), 16 Psoriatic Arthritis (PSA), 18 Rheumatoid Arthritis (RA) patients and 26 healthy controls (HC). Patient characteristics for the entire study cohort are summarized in Table 1. We studied the following inhibitors: Tofacitinib (hereafter abbreviated Tofa, reported inhibitory specificity JAK3 > JAK1/2), Ruxolitinib (Ruxo, JAK2 > JAK1), Baricitinib (Bari, JAK1/2 > TYK2) and CEP-33779 (CEP, JAK2). We additionally studied Bayer-18 (TYK2 > JAK2) but saw no consistent effects *in-vitro* in initial experiments. Therefore Bayer-18 was not investigated further. Concentrations of inhibitors were based on initial dose-response experiments using purified healthy control CD4+ T cells quantifying cytotoxicity and anti-proliferative capacity (Supplementary Fig. S1a–c). Figure 1a shows that multiple JAK inhibitors (Tofa, Ruxo, Bari and CEP) potently inhibited IL-17A production for inflammatory arthritis patients and controls (Original data are available in Supplementary Fig. S2a). No disease-specific effects were observed. In order to delineate the specific effect of the inhibitors on IL-23-triggered IL-17A production in AS, we tested the inhibitors on purified blood CD4+ T cells from AS patients in the presence of IL-2, or IL-2 + IL-23. Addition of IL-23 increased the production of Th17 cytokines. However, JAK-triggered inhibition of IL-17A and IL-22 secretion was greater for IL-2 stimulation compared to IL-2 + IL-23 stimulation, despite being significant in both conditions (p < 0.001, Supplementary Fig. S3a,b). Only Tofa inhibited IL-17A secretion similarly in IL-2, and IL-2 + IL-23 conditions. Effects on other Th17 cytokines and on IFNγ were assessed in a subgroup of AS patients and HC. Inhibition of IL-17F, IL-22 and IFNγ secretion was observed with multiple inhibitors, but only Tofa, Baricitinib and CEP inhibited Granulocyte-Macrophage colony-stimulating factor (GM-CSF) production significantly (p < 0.001, <0.05 and <0.001 respectively) in AS patients (Fig. 1b).

**Table 1:** Study population characteristics.

	HC (n = 26)	AS (n = 52)	PSA (n = 16)	RA (n = 18)
**age, years (median and range)**	40.1 [26;64]	41.0 [23;73]	47.9 [26;71]	53.6 [22;84]
**male/female, no**.	16/10	32/20	9/7	4/14
**HLA-B27 positive, %**	na	86.7^†^	50.0^†^	na
**RF positive, %**	na	na	na	75.0^‡^
**anti-CCP positive, %**	na	na	na	86.7^*^
**BASDAI (SD)**	na	4.6 (2.5)^+^	na	na
**DAS28 CRP (SD)**	na	na	na	2.7 (1.3)^^^
**CRP, mg/L (SD)**	na	18.4(26.9)^∞^	17.1 (27.5)^∞^	7.5 (9.9)^∞^
**Treatment**				
anti-TNF, no.	na	11/52	1/16	0/18
DMARD, no.	na	10/52	13/16	18/18
Steroid, no.	na	0/52	0/16	0/18
**Comorbidities**	na		na	na
Uveitis, no.		13/52		
Psoriasis, no.		4/52		
IBD (UC/CD), no.		6/52		

HLA-B27 (human leucocyte antigen-B27), RF (rheumatoid factor), anti-CCP (anti-cyclic citrullinated peptide), BASDAI (Bath Ankylosing Spondylitis Disease Activity Index), DAS28 CRP (Disease Activity Score 28 CRP), CRP (c-reactive protein), DMARD (Disease-modifying antirheumatic drug), IBD (inflammatory bowel disease), UC (ulcerative colitis), CD (Crohn’s disease).

^†^Data available for 44 AS and 4 PSA patients.

^‡^Data available for 16 RA patients.

^*^Data available for 15 RA patients.

^+^Data available for 45 AS patients.

^^^Data available for 14 RA patients.

^∞^Data available for 38 AS, 15 PSA and 17 RA patients.

### JAK inhibitors inhibit IL-17A production by established Th17 cells and by synovial fluid CD4+ T cells from AS/SPA patients

**Figure 2:**
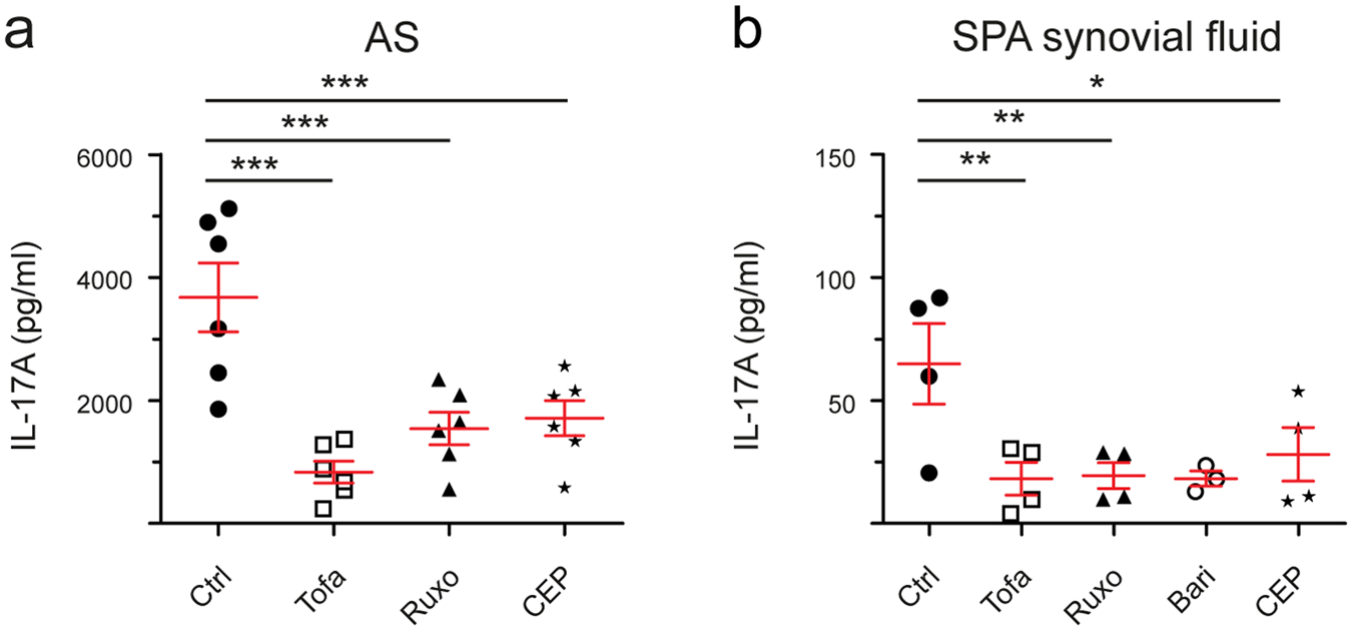
JAK inhibitors work on “type 17” cytokine production *in-vitro* in Spondyloarthritis on established peripheral Th17 cells and on synovial fluid CD4+ T cells. (**a**) Reduction of IL-17A secretion by JAK inhibitors (Tofa, JAK3 > JAK1/2; Ruxo, JAK2 > JAK1; Bari, JAK1/2 > TYK2; CEP, JAK2) in AS CD4+ T cells (n = 6), primed under Th17-promoting conditions for 6 days, upon restimulation with anti-CD2/3/28 beads for 24 hours measured by ELISA. **(b)** Effects of JAK inhibitors on IL-17A secretion (ELISA) from synovial CD4+ T cells of SPA patients cultured for 3 days (n = 4, Bari n = 3). Statistical analysis: mean ± SEM, repeated measures 1-way ANOVA followed by Dunnett’s method for multiple comparisons.

Addition of the inhibitors *in-vitro* also reduced IL-17A responses from established Th17 cell lines from AS patients (after 6 days of Th17-promoting conditions, Fig. 2a). Figure 2b shows that JAK inhibitors also effectively reduced IL-17A secretion by SPA synovial CD4+ T cells demonstrating effects on joint derived cells.

### Small molecule JAK inhibitors have broad inhibitory actions on STAT phosphorylation

**Figure 3:**
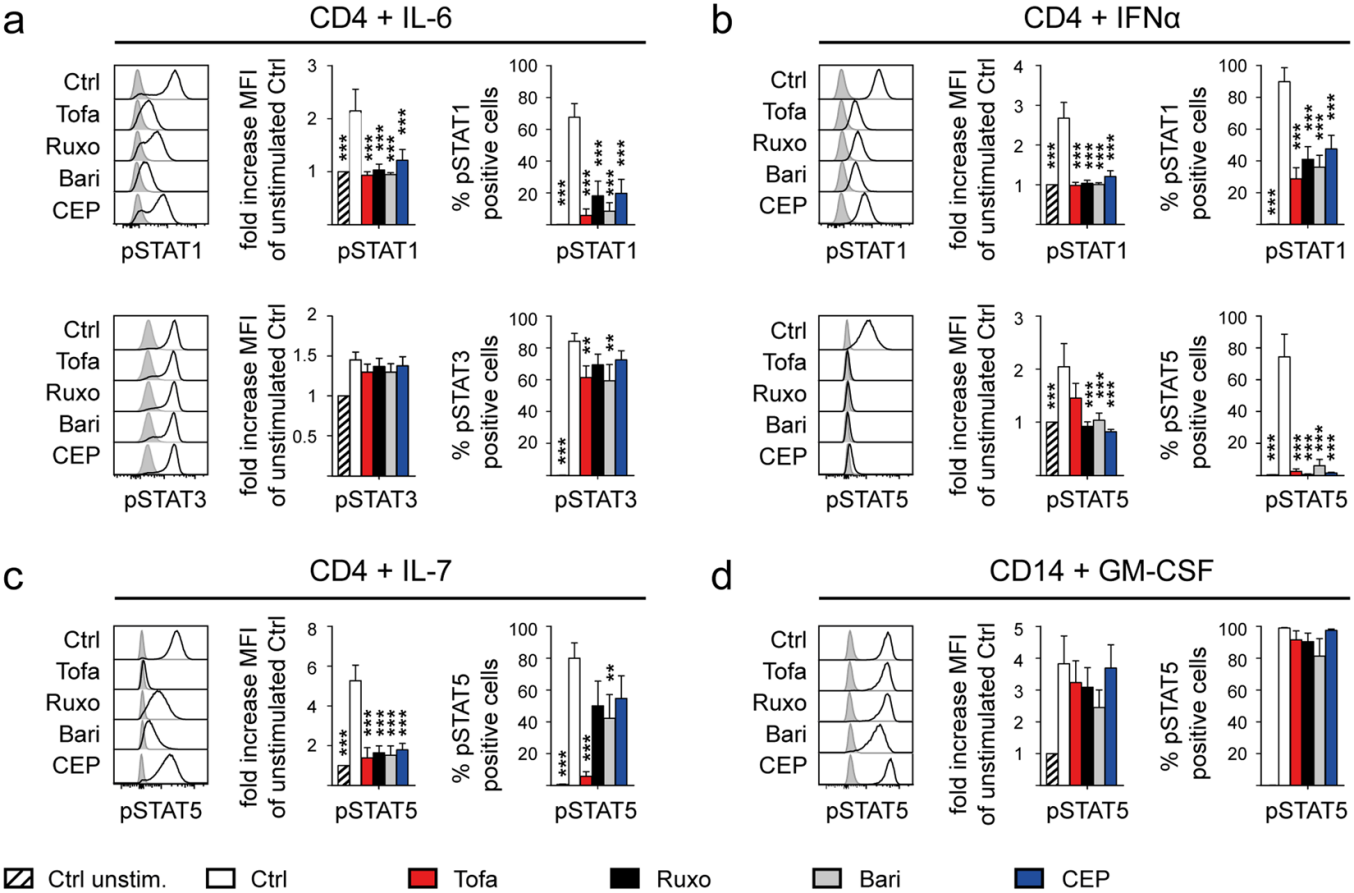
JAK inhibitors inhibit multiple cytokine-driven STAT phosphorylation events. Inhibition of STAT phosphorylation by JAK inhibitors (Tofa, JAK3 > JAK1/2; Ruxo, JAK2 > JAK1; Bari, JAK1/2 > TYK2; CEP, JAK2) in freshly isolated AS PBMC (n = 5–6) upon cytokine stimulation assessed by intracellular Flow Cytometry. Stimulation with **(a)** IL-6, **(b)** IFNα and **(c)** IL-7 gated on CD4+ T cells and with **(d)** GM-CSF gated on CD14+ Monocytes. Panel on the left shows exemplary flow cytometry plot (light grey filled curves in each panel show unstimulated control staining), middle panel shows fold increase of mean fluorescence intensity (MFI) compared to unstimulated control and right panel shows frequency of phosphorylated STAT of parental population. Statistical analysis: mean ± SEM, 2-way ANOVA followed by Bonferroni’s method for multiple comparisons.

We next analysed STAT phosphorylation in AS patient-derived PBMC upon stimulation with IL-6 (reported to signal through JAK1/2 – STAT1/3), Interferon α (IFNα; JAK1/TYK2 – STAT1/5), IL-7 (JAK1/3 – STAT5) and GM-CSF (JAK2 – STAT5), in the presence of Tofa, Ruxo, Bari and CEP (Fig. 3a–d and Supplementary Table S2). Following IL-6 stimulation, Tofa, Ruxo, Bari and CEP all inhibited STAT1 phosphorylation, but STAT3 phosphorylation was only modestly reduced by Tofa and CEP (Fig. 3a). Following IFNα stimulation signaling via STAT1 and STAT5 was equally affected by all inhibitors (Fig. 3b). IL-7-mediated STAT5 phosphorylation was most profoundly reduced by Tofa, less so by Bari, Ruxo and CEP (Fig. 3c). GM-CSF induced STAT5 phosphorylation (thought to be mediated via JAK2) was not significantly affected by any inhibitor (Fig. 3d). Thus the different inhibitors have broad but distinct inhibitory effects on phosphorylation of STAT1, 3 and 5 in our primary patient-derived cell-based assays.

### siRNA-mediated silencing of *JAK1* and *3* and *TYK2* inhibits type 17 cytokine responses

**Figure 4:**
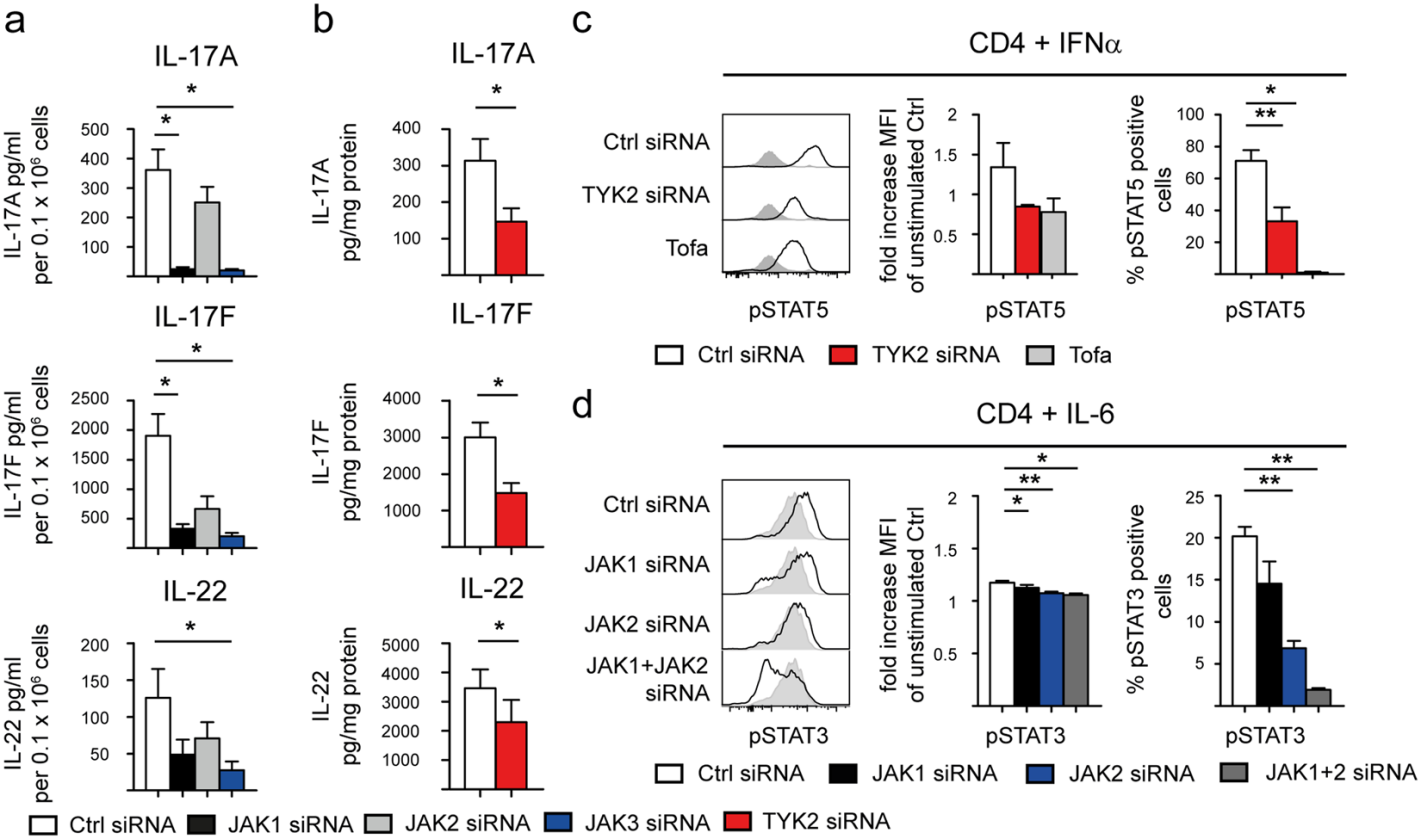
siRNA-mediated silencing of *JAK1 and 3 and TYK2* inhibits “type 17” cytokine responses. **(a)** Reduction of IL-17A, IL-17F and IL-22 secretion (ELISA) upon siRNA-mediated silencing of *JAK1/2/3* in HC CD4+ T cells cultured under Th17-promoting conditions for 3 days *in-vitro*. Data shown are normalized on cell number at the end of experiment = day 3 (n = 3–7). **(b)** Reduction of IL-17A, IL-17F and IL-22 secretion (ELISA) upon siRNA-mediated silencing of *TYK2* in HC CD4+ T cells cultured as in (a). Data shown are normalized on mg protein (n = 5/4/3). **(c)** Inhibition of STAT5 phosphorylation by siRNA-mediated *TYK2* silencing in HC CD4+ T cells upon IFNα stimulation 3 days post transfection compared to Tofa treatment (n = 2–5) and **(d)** of STAT3 phosphorylation by siRNA-mediated *JAK1* and *JAK2* silencing upon IL-6 stimulation (n = 1, triplicates). Panel on the left shows exemplary flow cytometry plot (light grey filled curves in each panel show unstimulated control staining), middle panel shows fold increase of MFI compared to unstimulated control and right panel shows frequency of phosphorylated STAT of parental population. Statistical analysis: mean ± SEM, paired t test.

In order to delineate the roles of the different JAK subtypes on “type 17” cytokine production we next determined the effects of siRNA-mediated silencing of *JAK 1-3* on Th17 responses in HC CD4+ T cells. We also silenced *TYK2*, as we had no specific TYK2 inhibitor included in our experiments so far. Efficient transfection and knock-down in HC CD4+ T cells was confirmed using labelled siRNA, Western Blot, flow cytometry and quantitative PCR (qPCR; Supplementary Fig. S4b–e). Targeting *JAK1* or *JAK3* inhibited IL-17A and IL-17F secretion, and *JAK3* silencing additionally reduced IL-22 secretion from HC CD4+ T cells cultured under Th17-promoting conditions for 3 days *in-vitro* (Fig. 4a). *TYK2* silencing also significantly reduced IL-17A, IL-17F and IL-22 secretion (p = 0.021/0.022/0.021, Fig. 4b), which was confirmed for IL-17A and IL-17F by qPCR (Supplementary Fig. S2f). siRNA-mediated silencing of *JAK2* had no significant effect in this assay (Fig. 4a). Subsequently we examined the effects of siRNA-mediated silencing of *JAK1*, *JAK2* and *TYK2* on STAT phosphorylation following IL-6 (reported to signal through JAK1/2 – STAT1/3) and IFNα (JAK1/TYK2 - STAT1/5) stimulation of HC CD4+ T cells, in order to clarify which JAK was the most promising target to interfere with Th17 maintenance and polarization. IFNα (effects thought to be mediated by JAK1/TYK2 and STAT1/5) was used as a surrogate for TYK2 specific signaling as IL-23 stimulation required preactivation of human CD4+ T cells for 5 days. Figure 4c shows that *TYK2* silencing indeed significantly inhibited STAT5 phosphorylation following IFNα stimulation as with Tofa treatment (p = 0.003 and 0.042). IL-6-induced phosphorylation of STAT3 was significantly reduced by *JAK2* silencing (p = 0.002, Fig. 4d). STAT1 phosphorylation upon IL-6 stimulation was significantly reduced by *JAK1* and *JAK2* silencing (p = 0.003 and 0.002, Supplementary Fig. S4h). Combination of *JAK1* and *JAK2* silencing showed an additive effect on the phosphorylation of STAT3 and 1 upon IL-6 stimulation.

## Discussion

We here describe potent *in-vitro* inhibition of CD4+ T cell production of three important “type 17” cytokines, IL-17A, IL-17F and IL-22, using both small molecule JAK inhibitors and siRNA-mediated gene silencing. Both approaches were effective for CD4+ T cells derived from patients with Ankylosing Spondylitis, Psoriatic Arthritis, Rheumatoid Arthritis as well as healthy controls.

Our data strongly reinforce the concept that compared to recombinant antibodies, which target only one or two cytokines at a time, a major advantage of JAK inhibition lies in the simultaneous inhibition of production of multiple Th17-related cytokines. As well as inhibiting IL-17F and IL-22, we here show additional inhibitory effects on GM-CSF production for Tofacitinib (JAK3 > JAK1/2) and CEP-33779 (JAK2) in AS CD4+ T cells. GM-CSF is likely an important pro-inflammatory cytokine in multiple inflammatory diseases including AS^23–25^. The moderate effects of the inhibitors on GM-CSF could possibly be attributed to the fact that GM-CSF single-producing lymphocytes may be more resistant to manipulation than other Th cell subsets, and/or that GM-CSF/IL-17A double producers contribute only modestly to overall GM-CSF production. Investigation of newer and more specific JAK inhibitors with regard to GM-CSF production by Th17 cells seems rational.

Another interesting aspect requiring more detailed investigation is the interference of JAK inhibition with “type 17” cytokine secretion from KIR3DL2+ (killer cell immunoglobulin like receptor, three Ig domains and long cytoplasmic tail 2) Th17 cells. These cells are enriched in HLA (human leucocyte antigen)-B27-positive SPA patients and contain the majority of IL-17A-producing CD4+ T cells^6^. These cells express higher levels of IL-23 receptor and upon binding of HLA-B27 heavy chain dimers produce increased amounts of IL-17A. The detailed effects of JAK inhibition on this KIR3DL2-B27 heavy chain dimer-induced IL-17A secretion remain to be investigated in another study.

Our data further suggest that, at least in our assays on primary patient-derived cells, current JAK inhibitors are only moderately selective for particular JAK family members and functionally target multiple cytokine pathways. One example for this moderate *in-vitro* selectivity is the fact, that CEP, with claimed 40-fold selectivity for JAK2 against JAK1, effectively blocks IFNα signaling in our assays, which is supposed to rely on JAK1 and TYK2. On the other hand CEP, as well as the other inhibitors, does not seem to block GM-CSF-induced STAT5 phosphorylation, which is JAK2-dependent. This could be explained by the fact that monocytes from patients with autoimmune disease show sustained STAT5 phosphorylation upon GM-CSF stimulation (greater than 24 h)^26^. This phosphorylation was found to be resistant to JAK2/3 inhibition by AG490, another small molecule inhibitor of JAK. These data suggest that STAT5 phosphorylation is not purely dependent on kinase activity, but could rather depend on mechanisms of dephosphorylation and recycling or degradation. JAK2 silencing on the contrary did lower but not significantly reduce IL-17F and IL-22 secretion. This can be explained by the smaller number of experiments (n = 3 compared to n = 7 for JAK1) and the relatively small population of IL-23 receptor-positive CD4+ T cells, in which *JAK2* silencing related effects might be overridden by IL-2. The specific effects of JAK inhibition on IL-23-induced STAT3 phosphorylation could not be examined in this study, as there was no robust shift if Il-23 was used on freshly isolated PBMC. This may be because IL-23-mediated STAT3 phosphorylation requires preactivation of PBMCs via CD3 and CD28 for 5 days or has to be examined in an IL-23 receptor expressing T cell line (e.g. kit225)^22,27^. IL-6 mediated phosphorylation of STAT3 was only modestly affected by small molecule inhibitors of JAK. This seems controversial, as STAT3 phosphorylation is the hallmark of IL-6 signaling^28^. However, it has been shown previously that IL-6 induced STAT1 phosphorylation is more sensitive to JAK inhibition than STAT3 phosphorylation and that this effect is dose dependent for Tofa. The dose used in our study is lower than the one previously reported to robustly inhibit STAT3 phosphorylation in human CD4+ T cells^18^. The observation that the effect of JAK inhibitors on Th17 cytokine secretion is mainly independent of IL-23 in our study could be explained by the relatively low percentage of IL-23R positive cells. As IL-2, predominantly signaling through JAK1 and JAK3, can expand established Th17 cells and induce IL-17 secretion from human PBMC, it is not surprising, that Tofa has a robust inhibitory effect on Th17 cytokine release in our assay^29^. Nevertheless our data showing robust inhibition of multiple Th17 cytokines (IL-17A, IL-17F and IL-22) under Th17-promoting conditions strongly support further clinical trials of JAK inhibitors in AS/SPA and other Th17-driven inflammatory diseases.

Surprisingly the adverse clinical effects of JAK inhibitors, which are mainly derived from clinical trials and from real world experience of Tofacitinib, have been relatively limited even though targeting multiple cytokine pathways^12^. Malignancy and infections are the major adverse effects observed with these inhibitors, but changes in lipid and serum transaminase levels, cytopenias, and a reduction of the glomerular filtration rate occur as well^12^. Reactivation of varicella zoster virus is one specific adverse effect of JAK inhibition; however disseminative disease is rare^30^. In regard of JAK selectivity sparing of JAK2 might avoid afore mentioned cytopenias. Reduced activity on JAK1 might ameliorate the effect of such inhibitors on antiviral responses, especially through Natural Killer cells^12^. As TYK2 is also involved in IL-10 and type I Interferon signaling, a selective TYK2 inhibitor might confer a risk for reduced intrinsic anti-inflammatory function and increased infections^12,31^. However, reports on human TYK2 deficiency show a less severe immunodeficiency phenotype compared to JAK3 deficiency^32,33^. So far safety profiles of JAK inhibitors cannot be accurately derived from the reported *in-vitro* function of the designated JAK. Long term observations and individual clinical trials will have to be undertaken to prove the superiority of selective inhibitors.

Although we were not able to test a small molecule inhibitor specific for TYK2, the efficacy of siRNA-mediated *TYK2* silencing on Th17-associated cytokine secretion supports strategies of TYK2 inhibition for the treatment of AS and other Th17-driven diseases (despite the above mentioned caveats and the observed discrepancy for IL-22 in protein versus messenger RNA level). Recent studies comparing human cell and tissue transcriptomes with proteomes have found that agreement between mRNA and protein levels occurs only in about 40%, with differing translational rates and posttranslational modifications accounting for these differences^34^. Despite difficulties encountered due to the conserved nature of the kinase domain, selective TYK2 inhibitors and novel approaches targeting the pseudokinase domain show promise^35,36^. Alternative pathways of targeting TYK2 by inhibitory peptides have also been employed^37^. Our silencing experiments also suggest that JAK1 and JAK2 might be effective targets for interference with Th17 responses. Baricitinib is a moderately JAK1-specific inhibitor that has already been approved for RA treatment in the USA and Europe^38^.

One further advantage of JAK inhibitors in regard to targeting multiple cytokine pathways might be an effect on the changes in bone metabolism observed in AS, namely syndesmophyte formation and bony ankylosis. Tofacitinib has been reported to inhibit both inflammation and new bone formation in murine Spondyloarthritis^39^. A current clinical trial of Tofacitinib in AS patients provides the first direct evidence that JAK inhibition might also ameliorate bone proliferation in humans^17^.

In summary our *in-vitro* data using patient-derived cells show effects of JAK inhibition on multiple “type 17” inflammatory cytokines in SPA, and strongly support further clinical trials of multiple JAK inhibitors of varying specificity in AS/SPA and other Th17-driven inflammatory diseases.

## Patients and Methods

### Patient samples

Heparinized venous blood (30 mL) was obtained from patients with AS (modified New York criteria), patients with PSA (classification of Psoriatric Arthritis criteria (CASPAR)), patients with RA (EULAR criteria) and HC with ethical permission (COREC 06/Q1606/139 and Oxfordshire Research Ethics Committee B 07/Q1605/35) and upon informed consent. Synovial fluid was obtained with informed consent and same ethical approval from Spondyloarthritis patients meeting assessment of spondyloarthritis international society (ASAS) criteria for axial spondyloarthritis^40^. Leukocyte cones were acquired from National Health Service Blood and Transplant. All research was performed in accordance with the relevant guidelines and regulations.

### Inhibitors

Tofacitinib, Baricitinib, Ruxolitinib, CEP-33779 and Bayer-18 were purchased from Selleckchem, CaymanChemical and Synkinase (reconstituted at 50 mM in DMSO and frozen in aliquots) and used at concentrations and durations described in Supplementary Table S1 immediately upon thawing.

### Cell Purification and Cell culture

Mononuclear cells from peripheral blood (PBMC) or synovial fluid (SFMC) were isolated by Ficoll density-gradient centrifugation (Histopaque; Sigma-Aldrich). 5 × 10^4^ negatively selected CD4+ T cells (Miltenyi Biotec, >90% purity on average) were cultured under Th17-promoting conditions as described in online Supplementary Material and Methods.

### ELISA

Supernatants were analyzed with ELISA kits (IL-17A, IL-17F, IL-22, IFNγ; ebioscience and GM-CSF; BioLegend).

### Cell viability and proliferation assessment

CD4+ T cells were labeled with 5 µM CFSE (Carboxyfluorescein succinimidyl ester; Molecular Probes) and cultured as above. Anti-Annexin V and 7-AAD (7-Aminoactinomycin D; Biolegend) staining was used for viability assessment after three days of *in-vitro* culture as above. Further information available in online Supplementary Material and Methods.

### Intracellular Flow Cytometry

Freshly isolated PBMC or CD4+ T cells were stimulated and stained for intracellular flow cytometry of STAT and JAK as described in online Supplementary Material and Methods and Supplementary Table S2.

### Silencing of *JAK1*, *JAK2*, *JAK3* and *TYK2*

CD4+ T cells were electroporated with 2 to 5 µM of the respective siRNA (Eurogentec and ThermoFisher Scientific) using the Neon™ Transfection System (ThermoFisher Scientific). See also online Supplementary Material and Methods and Supplementary Table S3.

### Statistical analysis

Between-group differences were determined as indicated in the figure legends using paired t-Test, repeated measures 1-way and 2-way ANOVA followed by Dunnett’s or Bonferroni’s method for multiple comparisons (GraphPad Prism software version 5). P values less than 0.05 were considered statistically significant.

### Ethics Approval

Oxfordshire Research Ethics Committee REC06/Q1606/139 and 07/Q1605/35.

## Electronic supplementary material

Supplementary File

## Data Availability

The datasets generated during and/or analysed during the current study are available from the corresponding author on reasonable request.
