# Supplementary material for: Inhibiting ex-vivo Th17 responses in Ankylosing Spondylitis by targeting Janus kinases
Source: Sci Rep. 2018 Oct 23;8:15645. doi: 10.1038/s41598-018-34026-1 (PMC6199284; doi:10.1038/s41598-018-34026-1)
Supplement: Supplementary file 1 — Supplementary File [file 41598_2018_34026_MOESM1_ESM.pdf]

SUPPLEMENTARY FILE

Inhibiting ex-vivo Th17 responses in Ankylosing Spondylitis by targeting Janus kinases

Ariane Hammitzsch, MD[a.b*]

Liye Chen, MD, DPhil[a]

Jelle de Wit, PhD[a]

M. Hussein Al-Mossawi, BmBch DPhil[a]

Anna Ridley, DPhil[a]

Takuya Sekine, PhD[a]

Davide Simone, MD[a]

Karen Doig[a]

Alla Skapenko, PhD[c]

Paul Bowness, MBBChir DPhil[a]

[a] Nuffield Department of Orthopaedics, Rheumatology and Musculoskeletal Sciences, University of Oxford, Windmill Road, OX3 7LD, Oxford, UK

[b] Department of Nephrology, Klinikum rechts der Isar, Technical University of Munich, Munich, Ismaninger Straße 22, 81675 Munich, Germany

[c] Division of Rheumatology and Clinical Immunology, Medizinische Klinik und Poliklinik IV, University of Munich, Pettenkoferstraße 8a, 80336 Munich, Germany

Current Address Jelle de Wit, National Institute for Public Health and the Environment (RIVM), Centre for Infectious Disease Control (CIb), Utrecht, Netherlands and Takuya Sekine, Unit for Hematology, Department of Medicine Huddinge, Karolinska Institutet, Stockholm, Sweden

***Corresponding author and reprint requests** Ariane Hammitzsch, Department of Nephrology, Ismaninger Straße 22, 81675 München, Ariane.Hammitzsch@gmail.com, 0049-89-41402231 (phone), 0049-89-41404878 (fax)

SUPPLEMENTARY MATERIALS AND METHODS

Inhibitors Tofacitinib, Baricitinib, Ruxolitinib, CEP-33779 and Bayer-18 were obtained from Selleckchem, CaymanChemical, Synkinase and reconstituted at 50 mM in DMSO, frozen in aliquots and used at concentrations and durations described in Supplementary Table S1 immediately upon thawing.

Cell Purification and Cell culture Mononuclear cells from peripheral blood (PBMC) or synovial fluid (SFMC) were isolated by Ficoll density-gradient centrifugation (Histopaque; Sigma-Aldrich). CD4+ T cells were negatively selected from PBMC or SFMC with a human CD4+ T-cell Isolation Kit (Miltenyi Biotec). The purity of this population was >90 % by flow cytometry. CD4+ T cells were cultured in conditions that promoted Th17 numbers and preserved Th1, Th2 and Treg numbers as follows: Roswell Park Memorial Institute medium (RPMI-1640; Sigma-Aldrich), supplemented with penicillin-streptomycin (50,000 units, 50 mg; Sigma-Aldrich), L-glutamine (2 mM; Gibco/Life Technologies), and 10 % fetal bovine serum (Sigma-Aldrich) at 37°C and 5 % CO_2 in a humidified atmosphere. Recombinant human IL-2 (100 IU/ml; Peprotech), IL-1β, IL-6 and IL-23 (all at 20 ng/ml; Peprotech), together with anti-CD2/3/28 beads (Miltenyi Biotec) at a ratio of 1 bead per 20 cells in 96-well plates (5 x 10^4 cells per well), for 3 d for cytokine analysis and cell viability assessment, and 6 d for proliferation analysis, was used unless stated otherwise.

ELISA Supernatants were analyzed on day 3 of culture with an IL-17A, IL-17F, IL-22 and IFNγ ELISA kit (ebioscience) and a GM-CSF ELISA kit (BioLegend).

Restimulation experiments CD4+ T cells of AS patients were cultured as described earlier for 6 days without the inhibitors. On day 6 0.25 x 10^6 live cells were restimulated for 24 h with anti-CD2/3/28 beads (Miltenyi Biotec) at a ratio of 1 bead

per 2 cells in RPMI-1640 containing 10 % FBS, penicillin-streptomycin (50,000 units) and L-glutamine (2 nM) in the presence of inhibitors. Supernatant was evaluated by ELISA as above.

Cell viability and proliferation assessment CD4+ T cells were labeled with 5 µM CFSE (Carboxyfluorescein succinimidyl ester; Molecular Probes) according to the manufacturer's instructions and cultured as above. Before flow cytometry, cells were additionally stained for surface markers (anti-CD4-APC and anit-CD8a-BV510; Biolegend) and viability (fixable viability dye eFluor780; ebioscience). Samples were analyzed on a LSRFortessa (BD Biosciences), and 2 x 10^4 live CD4+ T cells were recorded per sample. Data analysis was performed using FlowJo software (version X 10.0.7). For viability assays, cells were washed twice in cold PBS and stained with anti-Annexin V-APC and 7-AAD (7-Aminoactinomycin D; Biolegend) in Annexin V binding buffer (10x: 10 mM Hepes, pH 7.4, 140 mM NaCl and 2.5 mM $CaCl_2$) for 15 min at room temperature in the dark. Flow cytometry analysis was performed within 1 h of staining in Annexin V binding buffer.

Analysis of STAT phosphorylation by flow cytometry - Phosphoflow 0.5 x 10^6 freshly isolated AS patient PBMC per tested condition were incubated at 37°C and 5 % CO_2 for 20 min with Tofa, Ruxo, Bari, CEP and DMSO control in RPMI-1640. Cells were then stimulated for 15 min with IL-6 100 ng/ml, IFNα 40,000 IU/ml, IL-7 50 ng/ml and GM-CSF 50 ng/ml (all Peprotech) under the same conditions. Cells were then immediately put on ice and stained for surface markers (anti-CD3-BV780, anti-CD4-AF700, anti-CD8a-BV510, anti-CD19-BV421; BioLegend, anti-CD14-PE; Miltenyi Biotec) and viability (fixable viability dye eFluor780; ebioscience) in ice-cold FACS Buffer (PBS 1 % FBS) in the dark for 20 min. Cells were then fixed with

Fixation buffer (BD Biosciences, Cytofix™) at 37°C for 11 min and permeabilized with Perm Buffer III (BD Biosciences, Phosphoflow™) for 30 min. Staining of phosphorylated STAT was performed at RT for 1 h (anti-STAT1(pY701)-AF488, anti-STAT3(p Y705)-PE-CF594, anti-STAT5(pY694)-AF647; BD Biosciences, Phosphoflow™). Samples were acquired on a LSRFortessa (BD Biosciences) and analyzed using FlowJo software (version X 10.0.7).

Silencing of *JAK1*, *JAK2*, *JAK3* and *TYK2* 0.2 to 0.8 x10^6 live CD4+ T cells were stimulated with Phytohaemagglutinin (PHA) 2 µg/ml (Roche) for 20 h and then electroporated with 2 to 5 µM of the respective siRNA (Eurogentec and ThermoFisher Scientific, see online supplementary Table S3) using the Neon™ Transfection System (ThermoFisher Scientific). Cells were then cultured in RPMI-1640 supplemented with 10% FBS with 10 ng/ml IL-7 and 50 IU/ml IL-2 (both Peprotech) and anti-CD2/3/28 beads (Miltenyi Biotec) at a ratio of 1 bead per 5 cells for 24 h. Transfection efficiency (measured by flow cytometry using DY660-labeld siRNA (GE Dharmacon)) was > 90% on average. Cells were spun once and resuspended in RPMI-1640 supplemented with 10% FBS and 50 IU/ml IL-2, and 20 ng/ml of IL-1β, IL-6 and IL-23 (all Peprotech) for 48 h before analysis upon IFNα stimulation and in RPMI-1640 supplemented with 10% FBS and 50 IU/ml IL-2 and 10 ng/ml IL-7 for 48 h before analysis upon IL-6 stimulation. 72 h after transfection live CD4+ T cells were counted, used for Phosphoflow or lysed for Western Blot, and supernatant was collected for ELISA.

Western Blot CD4+ T cells were lysed in ice-cold RIPA Buffer (Cell Signaling Technology) containing Complete Protease Inhibitor (Santa Cruz Biotechnology) 72 hours post transfection as suggested by the manufacturer. Protein concentration of

the lysate was measured by BCA Protein Assay kit (Pierce™, ThermoFisher Scientific). SDS-Electrophoresis and protein transfer was performed after standard protocol. The following antibodies were employed: anti-TYK2 (D4I5T), anti-JAK2 (D2E12), anti-JAK3 (D1H3) and anti-rabbit-HRP (all Cell Signaling Technology). Substrate was from Millipore.

Intracellular flow cytometry of JAK1 0.4 x 10^6 CD4+ T cells were transfected with JAK1- or control-siRNA as described earlier and stained for intracellular JAK1 expression 72 h later. Cells were stained for surface markers (anti-CD3-BV786, anti-CD4-BV421, anti-CD8a-BV510; BioLegend) and viability (fixable viability dye eFluor780; ebioscience) for 20 min on ice. After Fixation with 4% Formaldehyde (Pierce™, ThermoFisher Scientific) for 10 min at 37°C cells were moved to ice for 1 min and washed. Cells were then permeabilized with -20°C cold 90% methanol on ice for 30 min and incubated for 1 h at RT with anti-JAK1 (Clone 73/JAK1, B; BD Biosciences) or a mouse IgG1κ isotype control (ebioscience). Incubation with anti-mouse-APC (BioLegend) was performed at RT for 30 min. Cells were then kept at 4°C overnight and analysed the next day (LSRFortessa (BD Biosciences) and FlowJo software (version X 10.0.7)).

Quantitative PCR RNA was isolated from CD4+ T cells 72 h post transfection using the RNeasy Plus Mini Kit (Qiagen). Reverse transcription of 200 ng RNA was performed using the High-Capacity cDNA Reverse Transcription Kit (ThermoFisher Scientific) following manufacturer's instructions. Gene expression was analysed using Taqman® probes (TYK2: Hs00177464_m1, JAK1: Hs01026983_m1, JAK2: Hs01078136_m1, JAK3: Hs00169663_m1, IL 17A: Hs00174383_m1, IL 17F: Hs00369400_m1, IL 22: Hs01574154_m1, RPL13A: Hs04194366_g1) and

TaqMan® Fast Universal PCR Master Mix on a ViiA™ 7 Real-Time PCR System (all ThermoFisher Scientific). Results were calculated on the ΔΔct-Method.

Statistical analysis Between-group differences were determined as indicated in the figure legends using paired t-Test, repeated measures 1-way ANOVA, and 2-way ANOVA followed by Dunnett´s or Bonferroni´s method for multiple comparisons (GraphPad Prism software version 5). P values less than 0.05 were considered statistically significant.

SUPPLEMENTARY TABLES

Supplementary Table S1 Reported specificity of inhibitors, used concentration in-vitro and exposure time per experiment.

		**Tofa^**	**Ruxo∞**	**Bari***	**CEP~**
**Relative IC50 (nM) †**	JAK1	112	3.3	5.9	40 fold
	JAK2	20	2.8	5.7	1.3
	JAK3	1	428	> 400	85
	TYK2	-	19	53	800 fold
**Concentration used in experiments (nM)‡**		250	50	50	100
**Length of exposure (h)**	Fig. 1a+b	72	72	72	72
	Fig. 2a	24	24	na	24
	Fig. 2b	72	72	72	72
	Fig. 3a-d	0.3	0.3	0.3	0.3
	Fig. 4c	0.3	na	na	na
	Fig. S1a+c	72	72	72	72
	Fig. S1b	144	144	144	144
	Fig. S2a	72	72	72	72
	Fig. S3a+b	72	72	72	72
	Fig. S4g	0.3	na	na	na

† in cell free enzyme-assay

‡ based on titration experiments on CD4+ T cells from healthy controls

^ Changelian PS et al. Science 2003 Oct 31;302(5646):875-8

∞ Quintas-Cardama A et al. Blood 2010 Apr 15;115(15):3109-17

* Fridman JS et al. J Immunol. 2010 May 1;184(9):5298-307

~ Stump KL et al. Arthritis Res Ther. 2011 Apr 21;13(2):R68

na: not applicable

Supplementary Table S2 Cytokine stimulation used, predicted JAK/STAT signaling pathways studied for STAT phosphorylation assays and inhibitors tested.

**Cytokine**	**IL-6**	**IFNα**	**IL-7**	**GM-CSF**
**JAK family member**	JAK1<br>JAK2	JAK1<br>TYK2	JAK1<br>JAK3	JAK2
**STAT family members**	STAT1<br>STAT3	STAT1<br>STAT3<br>STAT5	STAT5	STAT5
**Inhibitor**	***Ruxo***<br>***Bari***<br>Tofa<br>CEP	Bari<br>Ruxo<br>Tofa	***Tofa***<br>Ruxo<br>Bari	***CEP***<br>Ruxo<br>Bari<br>Tofa

Bold and italic indicates inhibitors most specific for the signaling pathway by manufacturer information.

Supplementary Table S3 siRNA sequences used

Target	siRNA sequence/code
TYK2[1]	5′-UUCUCAUGGACUGUCUUCAGAAUGG-3′ and<br>5′-GCAGCAAGUAUGAUGAGCAAGCUUU-3′
JAK1[1]	5′-GCACAGAAGACGGAGGAAAUGGUAU-3′ and<br>5′-GCCUUAAGGAAUAUCUUCCAAAGAA-3′
JAK2[2]	JAK2VHS41246
JAK3[3]	5′-CCAUGGUGCAGGAAUUUGU-3′ and<br>5′-GGGUCCUUCACCAAGAUUU-3′

[1] Koppikar P et al. Nature 2012 Sep 6;489(7414):155-9.

[2] Stealth siRNA, Invitrogen Life Technologies

[3] Gómez-Valadés AG et al. Mol Ther Nucleic Acids. 2012 Sep 4;1:e42

SUPPLEMENTARY FIGURES

Supplementary Figure S1

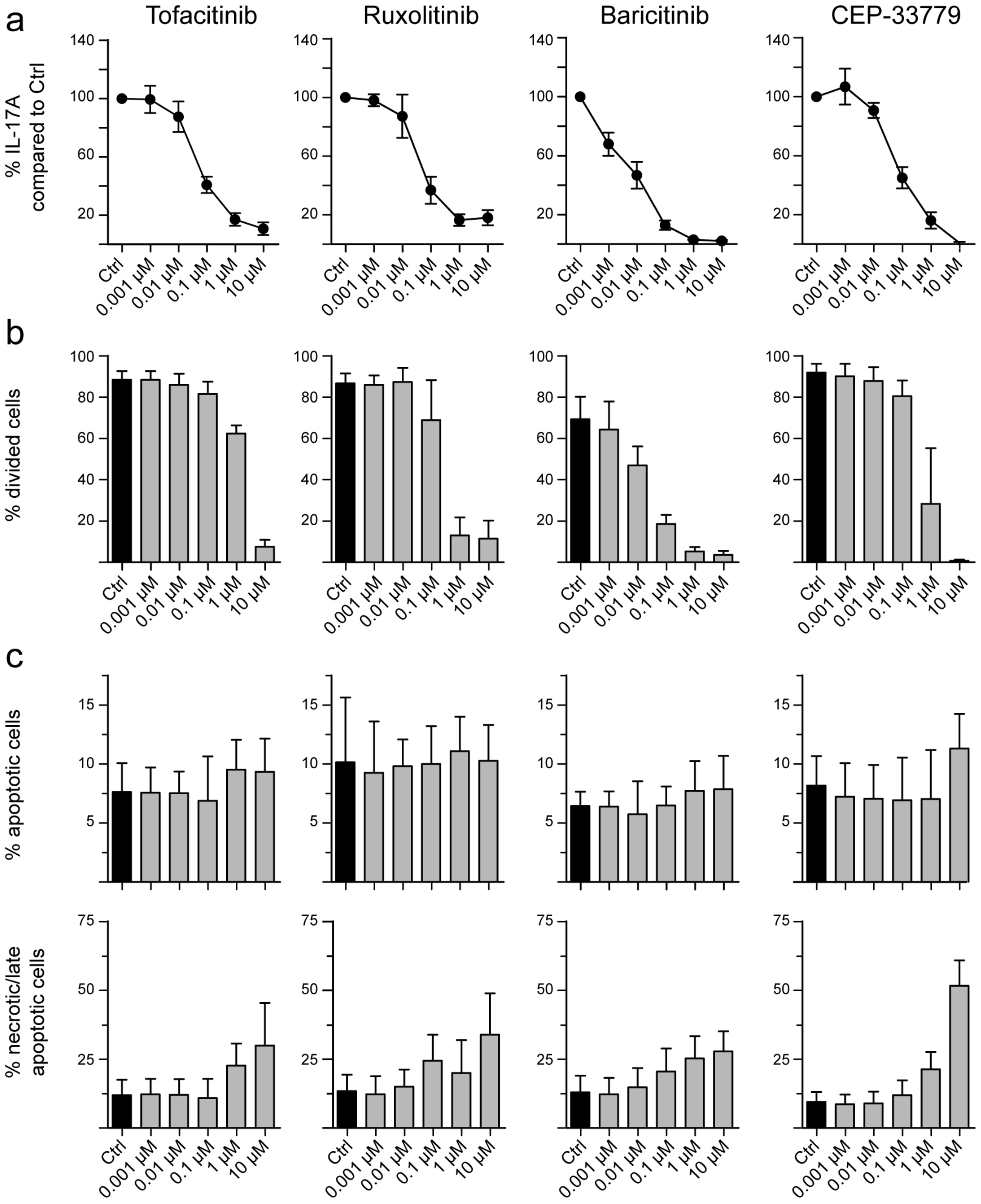

Supplementary Figure S1 Dose-dependent efficacy and toxicity of JAK inhibitors in-vitro.

(a) Dose-dependent inhibition of IL-17A secretion from HC CD4+ T cells kept under Th17-promoting conditions for 3 days in the presence of JAK inhibitors (Tofa, JAK3>JAK1/2; Ruxo, JAK2>JAK1; Bari, JAK1/2>TYK2; CEP, JAK2) in-vitro, measured by ELISA (n=4). **(b)** Anti-proliferative effects of JAK inhibitors (day 0-6) on CD4+ T cells assessed by CFSE-dilution on day 6 (n=4). **(c)** Toxicity of JAK inhibitors measured by flow cytometry and Annexin V/7-AAD staining on day 3 of in-vitro culture as in a (n=4).

Mean ± SEM are shown.

Supplementary Figure S2

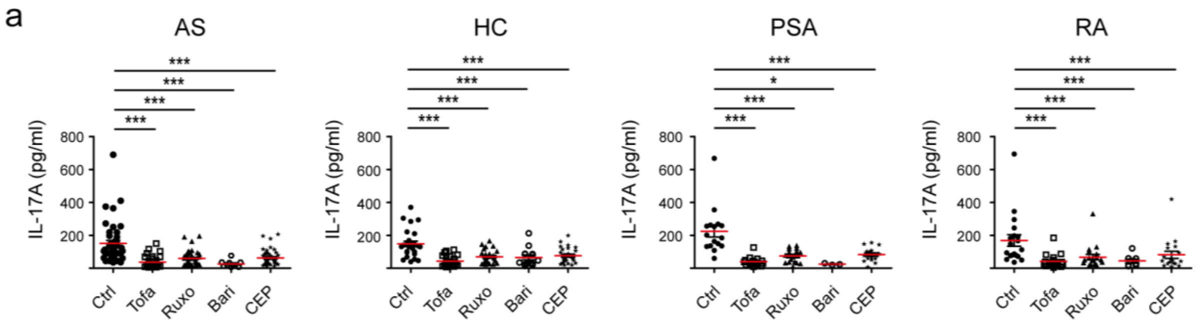

Supplementary Figure S2 JAK inhibitors inhibit CD4+ T cell type-17 cytokine production in-vitro in Spondyloarthritis, Rheumatoid Arthritis and healthy controls.

(a) IL-17A secretion from CD4+ T cells cultured under Th17-promoting conditions in-vitro in the presence of JAK inhibitors (Tofa, JAK3>JAK1/2; Ruxo, JAK2>JAK1; Bari, JAK1/2>TYK2; CEP, JAK2) from day 0. Measured by supernatant ELISA on day 3 (no.s AS=43/Bari=10, HC=26/Bari=14, PSA=16/Bari=3, and RA=18/Bari=9) and depicted in pg/ml.

Statistical analysis: mean ± SEM, repeated measures 1-way ANOVA followed by Dunnett´s method for multiple comparisons.

Supplementary Figure S3

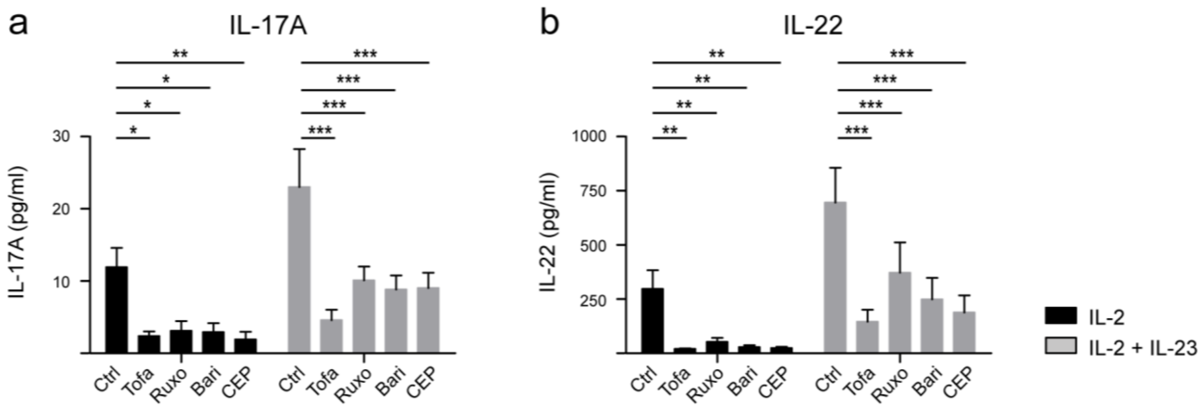

Supplementary Figure S3 Effects of JAK inhibitors on IL-23 triggered IL-17A and IL-22 secretion in peripheral blood CD4+ T cells of AS patients.

(a) IL-17A and **(b)** IL-22 secretion from AS patient CD4+ T cells cultured in the presence of IL-2 or IL-2 plus IL-23 under treatment with JAK inhibitors (Tofa, JAK3>JAK1/2; Ruxo, JAK2>JAK1; Bari, JAK1/2>TYK2; CEP, JAK2) from day 0. Measured by supernatant ELISA on day 3 (n=3).

Statistical analysis: mean ± SEM, repeated measures 2-way ANOVA followed by Dunnett´s method for multiple comparisons.

Supplementary Figure S4

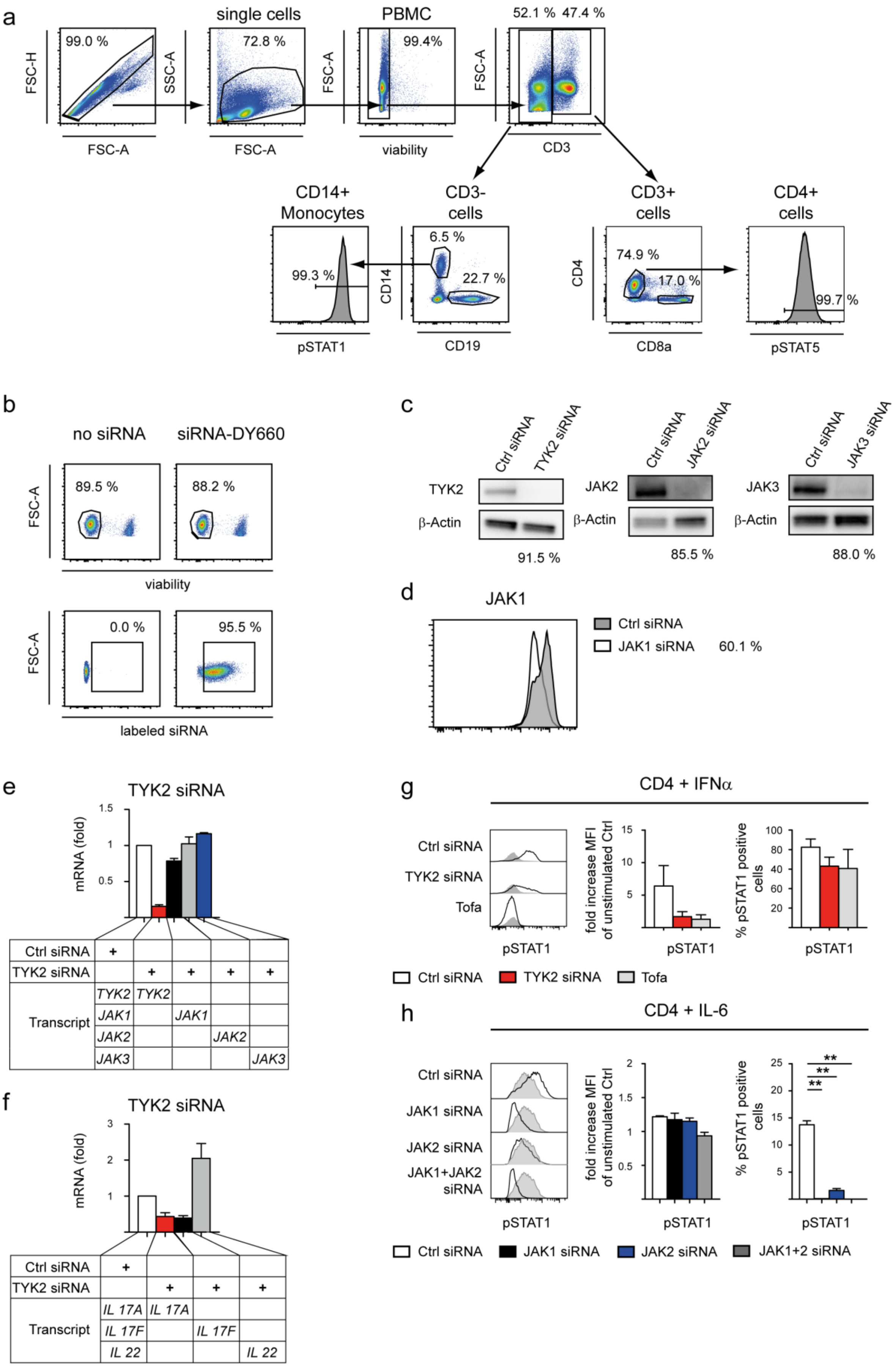

Supplementary Figure S4 Effects of siRNA-mediated silencing of different JAK family members in CD4+ T cells.

(a) Flow cytometry gating strategy for analysis of STAT phosphorylation upon cytokine stimulation in AS PBMC. Percentages represent the frequency of the parental population of the respective cell population. **(b)** Representative FACS plot showing transfection efficacy in HC CD4+ T cells after 24 hours. Top panel shows percentage of live cells and lower panel percentage of DY660-positive cells (=siRNA).

Efficacy of siRNA-mediated silencing of *TYK2*, *JAK2* and *JAK3* measured by Western Blot **(c)** and for *JAK1* measured by intracellular flow cytometry **(d)** in HC CD4+ T cells after 72 hours. Plots are representative of 5 (TYK2), 2 (JAK2), 3 (JAK3) and 1 (JAK1) experiments. Blots are cropped from different parts of the same gel for each silencing experiment in regard to JAK family member and loading control protein. For detailed exposure times and original blots see Supplementary Information.

(e-f) Silencing efficacy of TYK2-siRNA in HC CD4+ T cells after 72 hours measured by quantitative PCR of JAK family member transcripts **(e)** and of IL 17A, IL 17F and IL 22 **(f)**, and normalized to Control-siRNA (n=2). **(g)** Inhibition of STAT1 phosphorylation by siRNA-mediated *TYK2* silencing in HC CD4+ T cells upon IFNα stimulation 3 days post transfection, compared to Tofa treatment (n=2-5). **(h)** Inhibition of STAT1 phosphorylation by siRNA-mediated *JAK1* and *JAK2* silencing in HC CD4+ T cells upon IL-6 stimulation 3 days post transfection. Panel on the left shows exemplary flow cytometry plot (light grey filled curves in each panel show unstimulated control staining), middle panels shows fold increase of MFI compared

to unstimulated control and right panel shows frequency of phosphorylated STAT of parental population.

Mean ± SEM, paired t test.

Original Western Blots related to Supplementary Figure S2c

TYK2 and β-Actin

TYK2

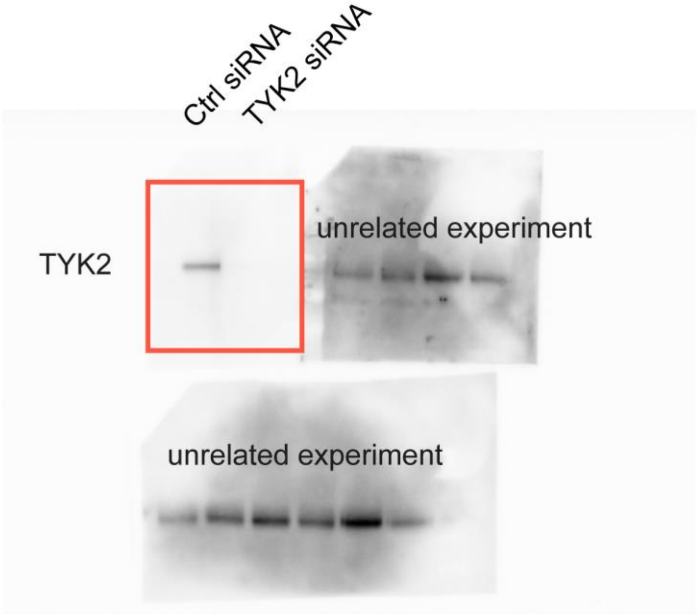

exposure 2min 30s

TYK2

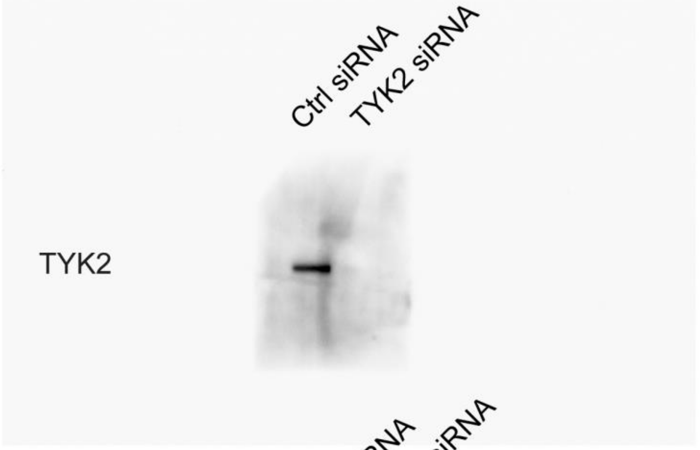

exposure 4min 17s

β-Actin

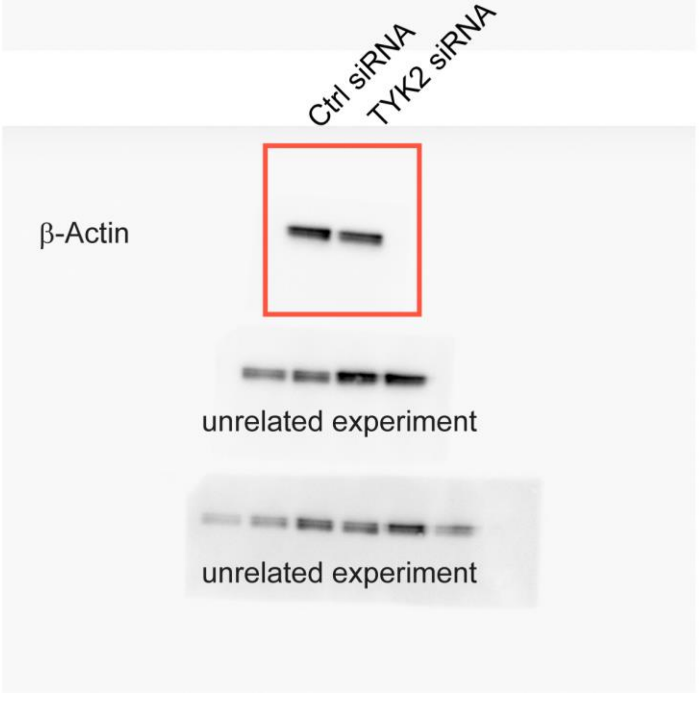

exposure 20s

Original Western Blots related to Supplementary Figure S2c

JAK2 and β-Actin

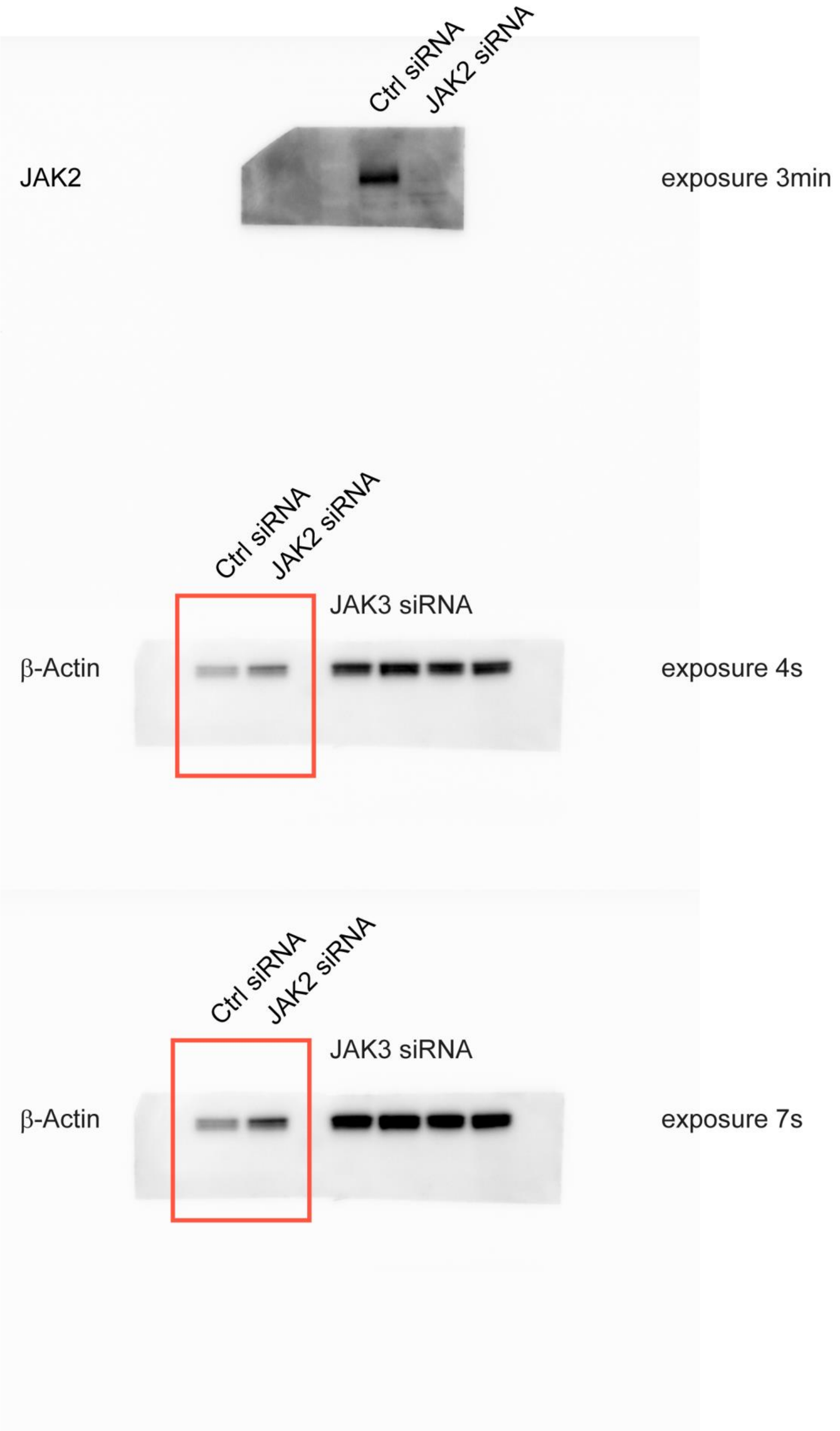

Original Western Blots related to Supplementary Figure S2c

JAK3 and β-Actin

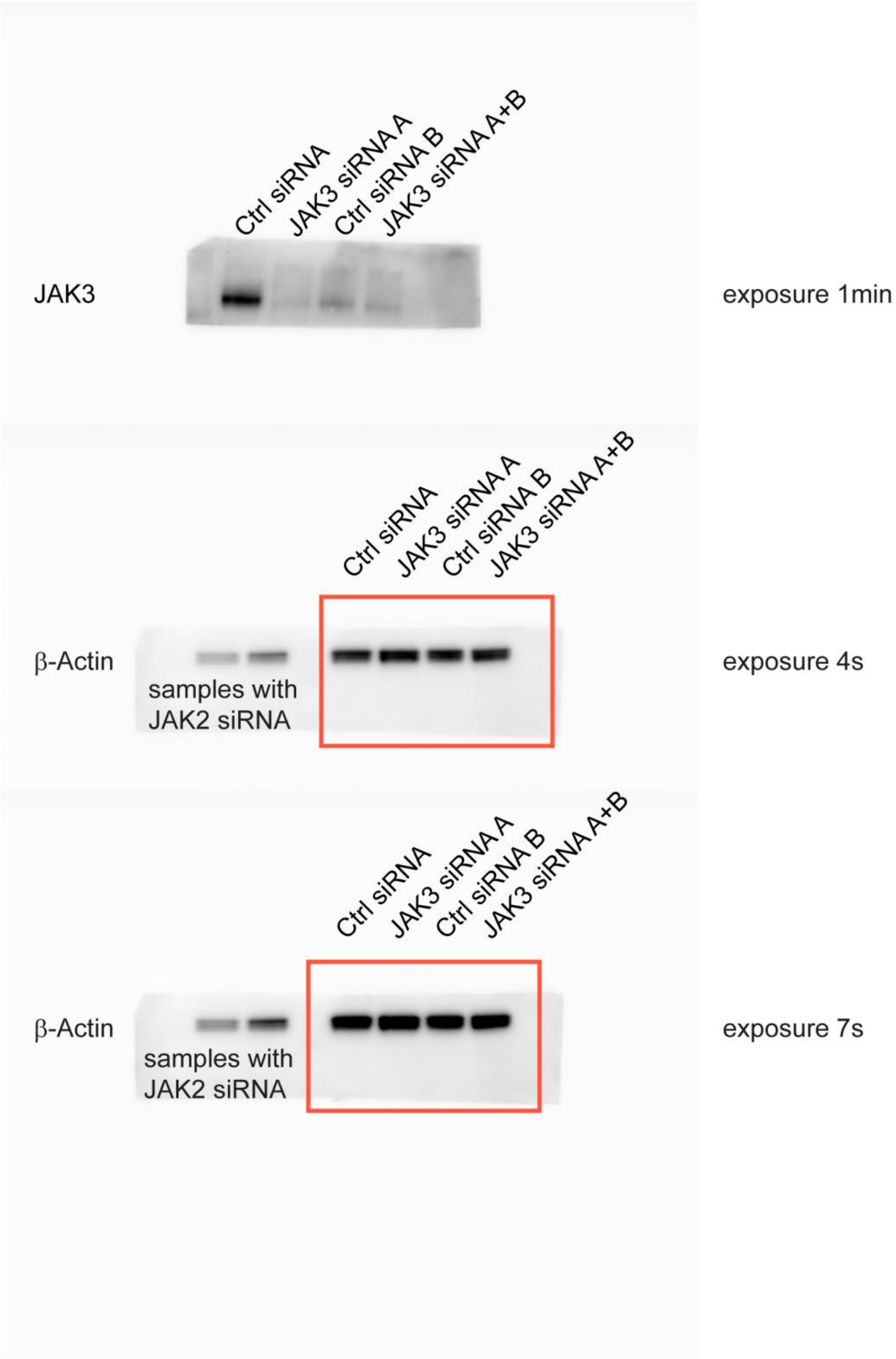

Of the different siRNA sequences tested initially (designated A and B), sequence A was chosen for the experiments.
